# Peptidylarginine Deiminases as Drug Targets in Neonatal Hypoxic–Ischemic Encephalopathy

**DOI:** 10.3389/fneur.2016.00022

**Published:** 2016-02-22

**Authors:** Sigrun Lange

**Affiliations:** ^1^Department of Pharmacology, UCL School of Pharmacy, London, UK; ^2^Department of Biomedical Sciences, University of Westminster, London, UK

**Keywords:** hypoxic–ischemic encephalopathy, peptidylarginine deiminases, deimination/citrullination, NETosis, microglia, microvesicles, epigenetics, microbiome

## Abstract

Oxygen deprivation and infection are major causes of perinatal brain injury leading to cerebral palsy and other neurological disabilities. The identification of novel key factors mediating white and gray matter damage are crucial to allow better understanding of the specific contribution of different cell types to the injury processes and pathways for clinical intervention. Recent studies in the Rice–Vannucci mouse model of neonatal hypoxic ischemia (HI) have highlighted novel roles for calcium-regulated peptidylarginine deiminases (PADs) and demonstrated neuroprotective effects of pharmacological PAD inhibition following HI and synergistic infection mimicked by lipopolysaccharide stimulation.

## Introduction

**Figure 1:**
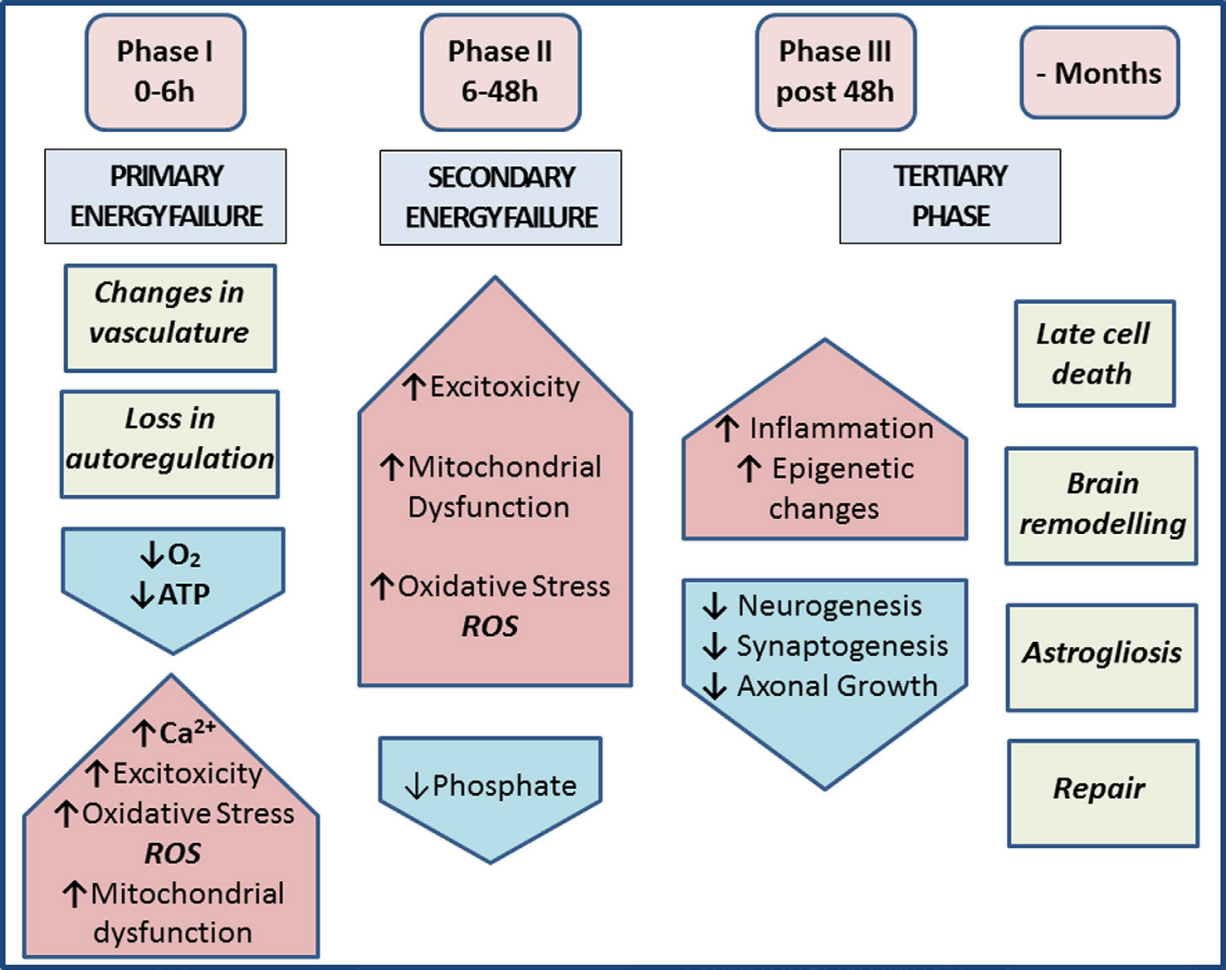
**Phases of injury post-HIE**. *Phase I* at 0–6 h post-insult includes vasculature changes and primary energy failure. This results in loss of autoregulation and severe lowering of the systemic arterial blood pressure. This causes decrease in oxygen, depletion of ATP, increased excitotoxicity, raised intracellular calcium, oxidative stress (ROS), and mitochondrial dysfunction. *Phase II* at 6–48 h post-insult and secondary energy failure lead to continued excitotoxicity, mitochondrial dysfunction, and oxidative stress; reduced phosphate levels. *Phase III* post 48 h shows injury to microglia, neurons, and astrocytes and also leads to continuous release of cytokines and other detrimental factors causing chronic inflammation, which in turn lead to epigenetic changes, as well as impairment of synaptogenesis, axonal growth, and neurogenesis. The tertiary phase continues months after injury involving late cell death, brain remodeling, and astrogliosis [based on Ref. (7, 17)].

Hypoxia and synergistic infection are major causes of perinatal brain injury (1–3), contributing to neurological disabilities affecting 1–8 cases per 1000 live births in the Western world and up to 26 per 1000 live births in underdeveloped countries (4, 5). In the acute phase of hypoxic–ischemic encephalopathy (HIE), cerebral blood flow is decreased, resulting in reduced oxygen and glucose delivery to the brain and anaerobic metabolism. Subsequent adenosine triphosphate (ATP) depletion leads to intracellular accumulation of sodium, water, and calcium due to reduced transcellular transport (6, 7). The cell releases excitatory glutamate upon membrane depolarization, allowing calcium to flow into the cell *via N*-methyl-d-aspartate (NMDA)-gated channels. The resulting peroxidation of free fatty acids by oxygen free radicals leads to more cellular damage. The culmination of energy failure, acidosis, glutamate release, lipid peroxidation, and toxic effects of nitric oxide (NO) leads to cell death *via* necrosis and apoptosis (7–9). The re-oxygenation stage following a neonatal hypoxic ischemia (HI) insult is followed by an intermediate “grace period” with little overt metabolic, nuclear magnetic resonance (NMR), or histological abnormalities, and only then by secondary energy failure, inflammation, apoptotic, necrotic and/or autophagic cell death, and axonal degeneration (10–13). During this intermediate phase, changes in cellular transcription, *de novo* protein synthesis, and posttranslational modifications all play pivotal roles (14–16). The tertiary phase of HIE during the months after the acute insult involves late cell death, remodeling of the injured brain, and astrogliosis (Figure 1).

Experimental studies in HIE animal models have shown roles for epigenetic mechanisms (18, 19), pH changes (20–22), and the tumor necrosis factor (TNF) gene cluster of cytokines in a combined inflammatory and HI brain insult (23). Clinical studies have shown protective therapeutic effects of hypothermia (24), which remains the standard care for HIE. Recent translational studies demonstrated significantly enhanced neuroprotection following co-therapy of hypothermia with xenon (25) or melatonin (26). Therefore, novel targets for synergistic therapies to enhance post-insult neuroprotection are of considerable interest.

## Peptidylarginine Deiminases

**Figure 2:**
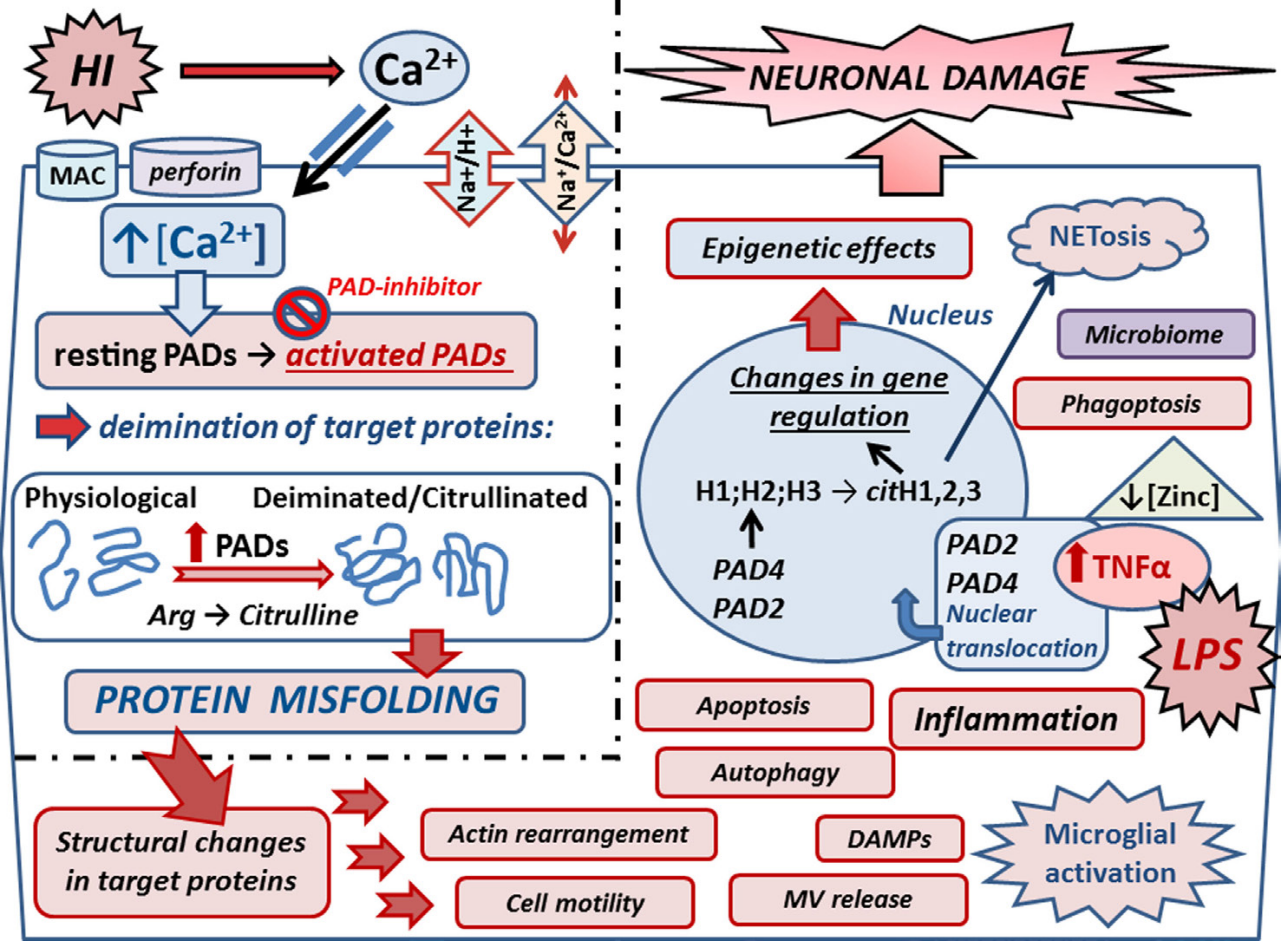
**Mechanisms of peptidylarginine deiminases (PADs) in HI and synergistic infection**. Upon HIE, Ca^2+^ entry is facilitated *via* reversal of the Na^+^/Ca^2+^ exchanger due to over activation of the Na^+^/H^+^ exchanger (NHE). Ca^2+^ entry can also be facilitated due to membranolytic pathways including the complement membrane attack complex (MAC) and perforin. Increased cytosolic Ca^2+^ triggers the neurotoxic cascade, which includes activation of the Ca^2+^-dependent PAD enzymes. Upon increased intracellular Ca^2+^, resting PADs are activated and catalyze the conversion of arginines into citrullines in target proteins. Protein deimination causes conformational changes and protein misfolding, affecting protein function and protein–protein interaction. Actin and cytoskeletal rearrangement is affected causing changes in cell motility, microvesicular (MV) release, autophagy, and phagoptosis. Deiminated neo-epitopes result in damage-associated molecular patterns (DAMPs) and contribute to inflammatory processes, such as microglial activation and downstream cascades. Inflammation causes upregulation of TNFα, which also is induced upon infection as mimicked by LPS stimulation in the HI/LPS synergy model. Raised TNFα levels cause translocation of PADs into the nucleus where they deiminate histones and affect gene regulation and epigenetics. Histone deimination also leads to extracellular trap formation (NETosis), which forms part of the microbial defense but can also cause the generation of neo-epitopes, leading to auto-inflammatory responses. Zinc is hypothesized to be a regulator of PADs, and as zinc levels are reduced in sites of infection, this regulatory role is lost, leading to increased protein deimination during infection. Individual variations of the neonatal microbiome may add to the complexity of the interplay of microbial defenses *via* NETosis and impact microglial activation and other mechanisms that remain to be elucidated. PAD inhibition with the pan-PAD inhibitior Cl-amidine has been successful in neuroprotection in the Rice–Vannucci mouse model of HI and HI/LPS synergy model, significantly reducing microglial activation, neuronal loss, and cell death. Refined inhibitors of PAD activation thus pose promising combinatory treatment options in synergy with current hypothermia measures.

The peptidylarginine deiminase (PAD) enzymes are a family of five calcium-dependent mammalian isozymes (PAD1, 2, 3, 4/5, and 6), located within a 355-kb gene cluster on human chromosome 1p36.13 (27–29) and mouse chromosome 4E1 with intron and exon boundaries well conserved throughout the evolution (27, 30). Each PAD isozyme has a unique tissue distribution pattern and substrate preferences (28, 31). PADs cause posttranslational protein deimination by modifying positively charged protein arginine residues into hydrophilic but uncharged citrullines, producing a loss of one positive charge *per* conversion and release of ammonia (30). The incidental disruption of ionic and hydrogen bonds within the substrate proteins affects protein structure, function, and protein–protein interactions (Figure 2). Main target proteins identified include nuclear histones (32), cytoskeletal, and structural proteins, such as components of the myelin sheath, intermediate filaments and associated adaptor proteins, extracellular components including fibrin and fibronectin (19, 33), and the chemokines (34–36). Intrinsically disordered proteins and β-turns are particularly prone to deimination (33). Deimination also has autoregulatory activity on PAD enzymes themselves and also upstream cytokines and chemokines, such as TNFα, C–X–C motif chemokine (CXCL) 8, and CXCL10 (37, 38). PADs are involved in physiological processes during development – including nerve growth and differentiation, gene regulation, regulation of pluripotency, and cell death (39–43). PAD dysregulation is associated with various inflammatory and degenerative diseases (30, 33, 34). PADs are also implicated in cancer (44–46), including through regulation of microvesicular (MV) release (47), which also is related to cerebral hypoxia induced by acute ischemic stroke (48, 49). PAD2 is considered the main central nervous system (CNS) isozyme (50), but PAD3 and PAD4 are also detected in neuronal tissue (19, 51, 52). PAD4 is the only isozyme with a designated nuclear translocation site, but the nuclear translocation of PAD2 and PAD3 has also been reported (19, 43, 44, 47). A novel role for PADs in acute CNS damage, and significant neuroprotective measures using pharmacological pan-PAD inhibition (Cl-amidine), was first described in a chick model of spinal cord injury (19). This neuroprotective effect was shown to be translatable to the Rice–Vannucci neonatal HI mouse model, resulting in significant protection of neuronal tissue and reduced microglial activation and cell death (53).

## PADs in Epigenetics

Epigenetic roles for PADs in gene regulation are supported in both HIE and HIE/lipopolysaccharide (LPS) synergy models where histone deimination, caused by PADs translocated to the nucleus, was abolished upon PAD inhibition. This paralleled the significant reduction observed in brain tissue loss, particularly in the cortex and hippocampus, which were the main areas affected (53). The LPS/HI model of infectious/ischemic form of brain damage is known to be strongly dependent on TNFα and related family of cytokines as global gene deletion of the whole TNF cluster of cytokines completely abolishes the synergistic, damage-enhancing effect of LPS on HI insult (23). In a transgenic multiple sclerosis murine model, exposure to elevated levels of TNFα-elicited nuclear translocation of PAD4 (54). In the same vein, PAD activity induced by HI insult and LPS stimulation increased neuronal damage following HI insult with beneficial effect of PAD inhibition. This indicates that LPS-induced upregulation of macrophage TNFα and related cytokines may act as upstream signals to PADs and histone deimination in HIE as well as in other forms of brain damage [(52); Figure 2].

## PADs in Phagoptosis

Microglia, the professional brain phagocytes, are strongly activated in HIE and can partly reduce inflammation by phagocytosis of dead/dying neurons and neuronal debris (9, 55–57). Microglia have also been shown to phagocytose live neurons, neutrophils, neural progenitors, and glioma cells causing death of the engulfed cell (58–62). Some phosphatidylserine (PS)-exposing neurons may be able to reverse their “eat-me” signals but still be phagocytosed *via* phagoptosis (62), which may dominate in conditions in which toll-like receptors (TLRs) are activated, as through LPS stimulation (58, 59). The roles of TLRs differ in the immature and adult brain following brain damage (63). Microglial activation can cause neurotoxicity by various other mechanisms, including NO generation by inducible NO synthase (iNOS), which inhibits neuronal mitochondria (64), or oxidant formation by phagocyte nicotinamide adenine dinucleotide phosphate (NADPH) oxidase, causing direct neurotoxicity (62, 65). In areas of ischemia and hypoxia, phagocytosis of dead or dying neurons may decrease inflammation by clearing harmful cellular components. Following mild ischemia, blocking phagocytosis could be beneficial by preventing the phagocytosis of stressed, but viable, neurons (59). Viable neurons in the post-ischemic brain are labeled by annexin V, indicating PS exposure that peaks at 3 days after transient ischemia and reverses thereafter (66), making these PS-exposed neurons targets for phagocytosis (59). The presence of phospholipids has been shown to reduce the calcium dependence of PAD2 by almost twofold *in vitro* (67), indicating that PAD activation may be facilitated in the presence of PS *in vivo*. In HIE, reduced microglial activation was observed 48 h post-insult in PAD inhibitor-treated animals in all brain regions and could be crucial for the protection of PS-exposed neurons against phagoptosis. This may be reflected in the significant reduction observed in neuronal loss in the PAD inhibitor-treated animals (53). Actin polymerization, facilitated by deimination and required for this mechanism, may be inhibited with PAD blockers as recently described in the regulation of cellular MV release (47). Actin, vimentin, and other cytoskeletal proteins were identified to be deiminated in acute CNS damage (19), while PAD3 inhibition has been shown to cause cytoskeleton disassembly in human neural stem cells as well as apoptosis-inducing factor (AIF) translocation to the nucleus (43). AIF has been shown to be a major contributor to neuronal loss induced by neonatal cerebral hypoxia-ischemia (68).

## PADs in Autophagy – Deiminated Neo-Epitopes

Autophagy is a cellular death mechanism recently implicated in HIE as a potential therapeutic target (69). This lysosomal pathway for intracellular degradation of macromolecules and organelles is important in the maintenance of cellular survival and homeostasis (70). Results are conflicting as pharmacological inhibition of autophagy was reported as neuroprotective (71), while studies inducing autophagy immediately after injury indicated it to be an endogenous neuroprotective mechanism (72). Recently, PAD4 was implicated in the induction of autophagy in hepatocellular carcinoma (73). Deiminated peptides were also shown to be processed in autophagy vesicles of antigen-presenting cells, indicating a linkage between autophagy and autoreactivity through the generation of deiminated neo-epitopes (74, 75), which in HIE could contribute to neuroinflammatory responses.

## PAD Activation via Membranolytic Pathways

Perforin and complement mediate membranolytic pathways of the cellular and humoral immune response and use a common “membrane attack complex component/perforin” (MACPF) domain to form pores in membranes to exert their potent anti-pathogen activity (76). Roles for both pathways have been described in the generation of hypercitrullinated proteins in rheumatoid arthritis (RA) (77). In a rodent study of HIE, the administration of complement C9 appeared detrimental (78) – which makes it tempting to speculate that hypercitrullinated proteins may be generated in HI in a similar fashion and contribute to microglial activation and neuroinflammatory damage.

Perforin oligomerizes in a Ca^2+^-dependent manner to form pores on target cells, triggering influx of calcium (79). In RA, this mechanism induced hypercitrullination in neutrophils, which express the PAD2, PAD3, and PAD4 isozymes, with each isozyme generating a distinct pattern of protein deimination (77). Again, it is tempting to speculate whether perforin contributes to neuroinflammatory responses in HIE.

## Zinc – A Regulator of PAD Activation?

Zinc is hypothesized to be an upregulatory modulator of calcium-dependent activation of posttranslationally acting PADs in autoimmune diseases (80). Zinc has previously been shown to inhibit the calcium-induced activation of PAD4 (81), and one can speculate that it may have a regulatory role for PADs in HIE. Putative links between zinc deficiency and HIE has been touched upon (82) in the context of effects of oxidative stress in childhood and neonatal disorders. The role of zinc may be particularly interesting with respect to HIE and synergistic infections as the zinc concentration is thought to be downregulated upon infection (80). This may cause downstream activation of the PAD cascade and further contributes to neuroinflammatory responses in HIE (Figure 2). Whether supplementing neonates with zinc as a preventive measure is open to debate.

## PADs in Neutrophil Extracellular Trap Formation (NETosis) – Interplay with the Microbiome

Recent studies have highlighted increasingly diverse roles for histone deimination including in NETosis, which forms part of the innate immune response in microbial defense and is implicated in autoimmunity (83). NETosis has recently received attention in neonatal infections (84) as neonatal neutrophils from umbilical cord were shown to differ significantly from adult controls in proteomic analysis and function including delayed NETosis (85). NETosis is dependent on the PAD4 isozyme (86, 87), which upon neutrophil activation, catalyzes arginine citrullination in three of the four core histones (88, 89). PAD4-null mice have been shown to fail to produce NETs, which coincides with lack of histone3 hypercitrullination (90). NETs are composed of DNA strands associated with antibacterial granular proteins, including deiminated histones, neutrophil elastase, myeloperoxidase, lactoferrin, and defensins (91). During inflammation, NETs can alert the immune system to danger by activating DNA receptors, such as TLR-9, helping in the recruitment of immune cells. TLR-9 activation *via* NETs in dendritic cells produces interferons, which can in turn initiate autoimmune responses (92, 93). Also, proteolytic and oxidative processing of proteins during NETosis can generate neoantigens (91), and NETosis has indeed been connected to several autoimmune diseases (92–97).

Neonatal neutrophils can form robust cellular aggregates and are able to release NETs in response to fungal hyphae when presented with extracellular matrix (84). In preeclampsia placentas, NETs have been shown to be in close contact with the syncytiotrophoblast, putatively obstructing the intervillous space, reducing blood flow, and leading to hypoxia in the fetus (98). NETosis can also be induced by activated endothelial cells and cause damage of the endothelium leading to more severe preeclampsia (99).

In the murine HIE/infection synergy model, LPS stimulation prior to neonatal HI insult may activate NETosis, which can result in further damage of surrounding tissue – emphasizing that this innate defense mechanism may be a double-edged sword when toxicity is directed against own tissues (53). It is interesting to note that PAD inhibitor effectively reduced histone deimination, which coincided with significantly reduced neuronal damage and cell death (53). As NET formation has been shown to require functional tubulin and actin filaments (100) and PAD blocker inhibits actin and tubulin deimination (19, 47), this may affect the ability of NET formation.

The capability of premature and healthy term infant cord neutrophils to form NETs is based on material from cords donated after cesarean section (84, 86). Labor deliveries are reported to increase the surface expression of TLRs on monocytes, and interleukin 8-induced neutrophil chemotaxis has been shown to be enhanced due to the stress of birth (101). As the effect of the microbiome in health and disease is gaining ever increasing attention, differences in the maternal and neonatal microbiome are emerging as crucial in response to infection, development, and neurological developmental disorders (102–104). It is tempting to speculate how individual differences in the neonatal microbiome may affect the capability of dealing with infections *via* mechanisms, such as NETosis – also in the wider context of cesarean sections versus vaginal delivery and breastfeeding – both of which influence the microbial colonization of the gut and the composition of the microflora (105, 106). This is particularly interesting with respect to emerging evidence of the importance of the neonatal gut–brain axis (107), a link between microglial maturation and the microbiome (108), sexual dimorphism in microglial numbers and response to HIE (109), and the connection between the maternal microbiome and pregnancy outcomes, including preterm births (110). Future studies comparing the effect of cesarean section versus labor delivery on the neonatal response to fungal infections and other pathogens will provide better understanding of the role of NETosis and PADs in neonatal HIE and synergistic infections.

## pH Changes and PAD Activity

Brain intracellular pH (pHi) is implicated in HIE (20–22). The Na^+^/H^+^ exchanger, which regulates pHi and cell volume, is over-activated during HIE, resulting in Ca^2+^ entry *via* reversal of the Na^+^/Ca^2+^ exchanger. Increased cytosolic Ca^2+^ triggers the neurotoxic cascade, which includes activation of the Ca^2+^-dependent PAD enzymes and downstream protein deimination (Figure 2). Activation of the Na^+^/H^+^ exchanger also leads to rapid normalization of pHi and to an alkaline shift. Brain pHi changes are closely involved in the control of cell death after injury as alkalosis enhances excitability, while a mild acidosis has the opposite effect (21). The degree of brain alkalosis in 78 babies with neonatal encephalopathy was related to the severity of brain injury (21). The effect of pH on PAD-catalyzed reaction and its kinetics has been studied to some extent (81). PAD4 has pKa values of ~6.2 and ~7.9, the pH optimum being pH 7.6, while calcium dependence of PAD4 is essentially pH-independent over the range of pH 6.0–8.5 (81). The PAD activation pathway should thus be stable over a relatively wide pH range.

## Hypothermia and PAD Inhibition – A Synergistic Treatment Option

Cooling brain or whole body to 33°C (moderate hypothermia) is currently the only strategy with a clearly demonstrated clinical benefit in babies with HIE. Therapeutic hypothermia promotes neuroprotection by several mechanisms including a decrease in intracellular calcium influx, which may improve serum Ca^2+^ levels and homeostasis (111–114). Excessive Ca^2+^ influx during and after a significant HI insult promotes depolarization of the mitochondria and abnormal excitatory receptor activity promoting further Ca^2+^ entry into the cells (113). Post-insult hypothermia suppresses this intrinsic pathway (114) and should also affect the Ca^2+^-regulated PAD activation pathway as calcium binding to PAD4 promotes the bioactive conformation, increasing PAD4 activity by 10,000-fold (115). The effects of cooling on calcium levels was investigated in a piglet model where plasma calcium increased to levels compared with normothermic animals within 3 h of hypothermia and could thus aid Ca^2+^ homeostasis in HIE (116). As several injury-associated signals, e.g., phosphorylated extracellular signal-regulated kinases (ERK), are increased following hypothermic intervention (117), combined therapies are desirable for increased neuroprotective effects. Hypothermia has been combined with adjunct therapy including xenon, erythropoietin, melatonin, and stem cells for substantial neuroprotection (118–125).

The success of reducing brain injury responses with pharmacological PAD inhibition in murine mouse models of HIE is promising as the treatment proved translatable between both animal and neuronal injury models. The effects observed on changes in histone deimination suggest a role for epigenetic regulation in response to CNS injury, while changes in deimination of cytoskeletal components could affect apoptosis, cell motility, and the ability of injured neurons to regrow axons (19, 43, 53). While the pan-PAD inhibitor Cl-amidine (126) still remains the most used experimental inhibitor to date, the therapeutic potential and generation of selective PAD inhibitors is receiving ever increasing attention (52, 127–131).

## Conclusion

A novel role for calcium-dependent PAD is highlighted in neural impairment in neonatal HIE, emphasizing their role as promising new candidates for drug-directed intervention in neurotrauma. During hypoxic ischemic insult, PADs are activated due to calcium dysregulation and putative upregulatory changes in zinc balance, which is decreased upon synergistic infection. Deiminated proteins are irreversibly modified due to changes of protein-bound arginines into citrullines, leading to protein misfolding and changes in protein function. In the case of synergistic infection, TNFα is upregulated and causes nuclear translocation of PAD enzymes, resulting in histone deimination and downstream changes in gene regulation. Mechanisms of PADs in HIE also touch upon the interplay of the microbiome, cesarean versus vaginal deliveries, and how neutrophil and microglial responses to pathogens could be affected. Isozyme-specific PAD inhibition through refined pharmacological intervention poses a promising combinatory treatment option in synergy with current head-cooling approaches.

## Author Contributions

The author confirms being the sole contributor to this manuscript and approved it for publication.

## Conflict of Interest Statement

The author declares that the research was conducted in the absence of any commercial or financial relationships that could be construed as a potential conflict of interest.
